# Ancestry-specific genetic effects on urinary 6-sulfatoxymelatonin: a multi-ancestry GWAS meta-analysis

**DOI:** 10.21203/rs.3.rs-7723851/v1

**Published:** 2025-10-12

**Authors:** Magdalena Żebrowska, Ziwei Zhang, Gwo-Tsann Chuang, Daniel S. Evans, Jesse Valliere, Matthew Maher, Jie Hu, Rebecca Richmond, Constance Turman, Jaime E. Hart, Jacqueline Lane, Loic Le Marchand, Lynne Wilkens, Matthias Wielscher, Christopher Haiman, Iona Cheng, A. Heather Eliassen, Katie L. Stone, Gregory J. Tranah, Yi-Cheng Chang, Lorelei Ann Mucci, Eva S. Schernhammer, Richa Saxena

**Affiliations:** Medical University of Vienna; Harvard T.H. Chan School of Public Health; National Taiwan University Hospital, National Taiwan University; California Pacific Medical Center; Massachusetts General Hospital; Massachusetts General Hospital; Harvard T.H. Chan School of Public Health; University of Bristol; Harvard T.H. Chan School of Public Health; Harvard Medical School; Massachusetts General Hospital; University of Hawaii Cancer Center; University of Hawaii Cancer Center; Medical University of Vienna; University of Southern California; University of California, San Francisco; Harvard Medical School; California Pacific Medical Center; California Pacific Medical Center; National Taiwan University; Harvard T.H. Chan School of Public Health; Medical University of Vienna; Harvard Medical School

**Keywords:** melatonin, sulphatoxymelatonin, GWAS, meta-analysis, multi-ancestry

## Abstract

Melatonin regulates circadian rhythms, metabolism, and immunity. Its primary metabolite, 6-sulfatoxymelatonin (aMT6s), is a biomarker linked to cancer risk and metabolic disorders. However, genetic determinants of aMT6s remain poorly understood, with only one prior GWAS limited to an East Asian cohort.

We conducted the first multi-ancestry genome-wide association meta-analysis of urinary aMT6s, integrating 11,744 participants from five cohorts: East Asians (Taiwan Biobank), European women (Nurses’ Health Studies), European men (MrOS), and multiethnic participants (MEC). aMT6s was measured from overnight or first-morning urine samples. Analyses used MR-MEGA and fixed-effects models in METAL. Polygenic risk scores (PRS) were constructed with PRS-CSx and tested for phenome-wide associations in the Mass General Brigham Biobank and UK Biobank.

No genome-wide significant loci were identified, and previously reported East Asian signals were not replicated. At suggestive significance, 23 loci emerged, with eight supported by both MR-MEGA and METAL. Two loci (SLIT3 rs1875972 and C12orf55 rs7137724) showed ancestry-specific heterogeneity, underscoring the role of population context. PRS analyses revealed robust associations with type 2 diabetes and sleep duration, linking aMT6s genetics to metabolic and circadian traits.

These findings highlight context-dependent genetic architecture of melatonin metabolism and emphasize the importance of ancestry in interpreting biomarker GWAS.

## Introduction

Melatonin, a hormone synthesized by the pineal gland, plays a critical role in regulating circadian rhythms, cellular metabolism, and immune responses. Its primary metabolite, 6-sulfatoxymelatonin (aMT6s), measured in overnight or first morning urine, serves as a stable and reliable biomarker for assessing nocturnal melatonin secretion in clinical and research settings^1^. This metabolite exhibits profound physiological and pathological associations. Elevated nocturnal urinary aMT6s levels, for instance, are positively correlated with tumor cell proliferation in gastrointestinal and lung cancer patients^2^, while reduced levels in renal transplant recipients are linked to higher mortality, underscoring melatonin’s potential therapeutic relevance^3^. Low levels of aMT6s are also associated with increased risks of breast^4–6^, oral^7^, gastric^8^ and prostate cancer^9,10^. Additionally, variations in melatonin patterns, such as the timing of dim light melatonin onset (DLMO), offer valuable insights into its temporal dynamics and their connections to chronotype and aging^11^. In people with Rare Genetic Neurodevelopmental Disorders (RGND), irregularities in melatonin production play a role in circadian rhythm disruptions and associated sleep difficulties^12^. Melatonin metabolism is influenced by both environmental and genetic factors. Night-shift work, for example, can suppress melatonin production^13^ while genetic variations, such as the rs10830963 G allele in the MTNR1B gene, have been associated with altered melatonin signaling and an increased risk of type 2 diabetes mellitus. Disruptions in melatonin rhythms, such as those observed during night-shift work, can lead to secretion patterns that worsen metabolic dysregulation^14^.

Despite its significance, our understanding of genetic determinants of melatonin secretion remains limited. A recent study in 2,373 East Asian ancestry individuals from the Taiwan Biobank did not identify any genome-wide significant loci, and reported five suggestive (p < 5·10^−6^) loci associated with urinary aMT6s^15^. Genetic variation, however, exhibits significant differences across ethnic groups, often resulting in population-specific effects. Therefore, the absence of large-scale genome-wide association studies (GWAS) of the melatonin metabolite together with limited racial and ethnic diversity in existing research represent critical limitations. This gap restricts the generalizability of findings and impedes a comprehensive understanding of melatonin’s role in health across diverse populations. Furthermore, to the best of our knowledge, currently available datasets that simultaneously include comprehensive genetic information and aMT6s measurements lack sufficient sample sizes to achieve the statistical power necessary for robust genetic studies, thereby hindering advancements in this area of research. To address these limitations, we aggregated data from the Taiwan Biobank (TBB), the Nurses’ Health Study I (NHS1), the Nurses’ Health Study II (NHS2), Osteoporotic Fractures in Men (MrOS), and the Multiethnic Cohort (MEC). By pooling urinary aMT6s data and genetic information across these cohorts, we aimed to perform a GWAS meta-analysis that increases statistical power and incorporates diverse populations.

## Results

Among the 11,744 individuals (54.5% men), genetically determined ancestry assignments identified 6,925 (59%) with European, 2,373 (20.2%) with East Asian, 1,494 (12.7%) with Japanese, 426 (3.7%) with African, 290 (2.5%) with Native Hawaiian and 226 (1.9%) with Latino ancestry. The average age of participants was 62.3 years (SD_age_=10 years) with MrOS cohort containing the oldest (mean_age_=73.1 (SD_age_=5.6)) and Taiwan Biobank representing the youngest cohort (mean_age_=50.8 (SD_age_=10.8); Table 1).

### Cohort specific heritabilities and genetic correlations

We estimated cohort-specific heritabilities from cohorts’ GWAS summary statistics, filtered to include only high-quality SNPs from the HapMap 3 reference panel^25^. Heritabilities ranged from low for the NHS cohort (h^2^_NHS_ = 0.1582 (SE_NHS_=0.1683)) to moderately high for MrOS (h^2^_MrOS_ = 0.4182 (SE _MrOS_=0.2666)) (Supplementary Table A1). LDSC regression analysis indicated positive genetic correlation between NHS and MrOS (r_g_=0.8073), moderate positive correlation between NHS and MEC (r_g_=0.5316) and weak negative correlation between MEC and MrOS (r_g_=−0.0085), but all with large standard errors relative to estimates themselves, and insignificant p-values (p > 0.17) (Supplementary Table A2).

### Meta analysis

**Figure 1.**
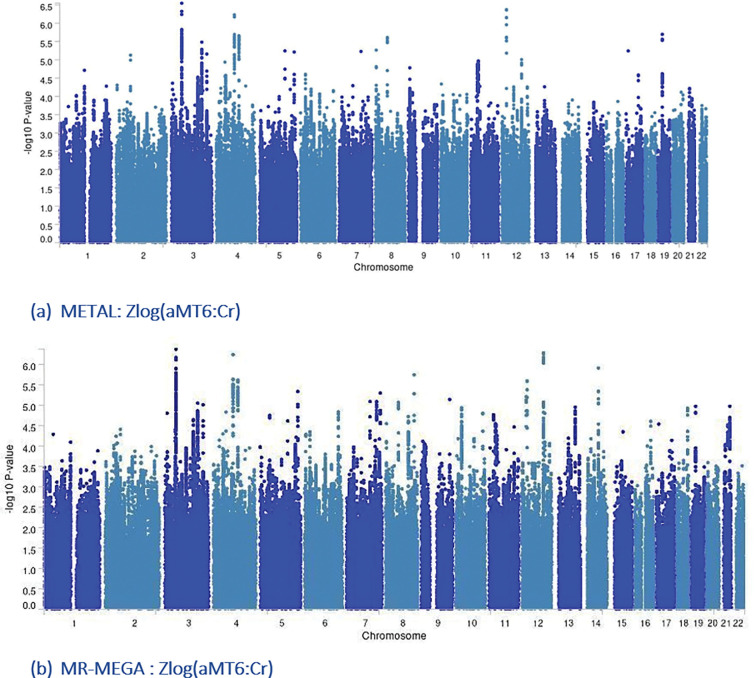
Manhattan plots of the GWAS meta-analysis results across 11,744 study participants obtained using METAL inverse variance fixed-effects model with genomic control correction or MR-MEGA meta-regression test for Zlog(aMT6:Cr). The x-axis represents chromosomes and base pair positions of variants tested in the meta-analysis, while the y-axis shows −log_10_ p-values from the two-sided variant association test.

**Figure 2.**
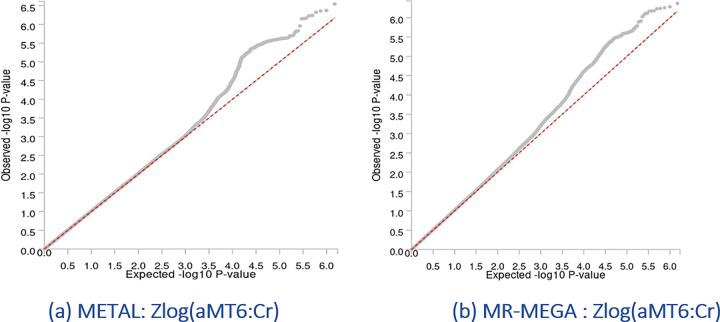
QQ plots of the GWAS meta-analysis results for Zlog(aMT6:Cr) across 11,744 study participants (a) METAL inverse variance fixed-effects model with genomic control correction; (b) MR-MEGA meta-regression test.

**Figure 3.**
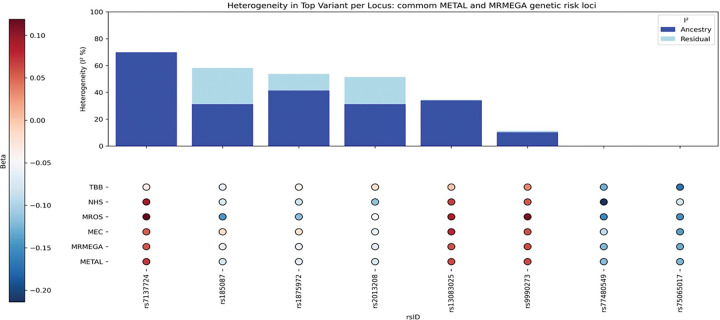
Heterogeneity in lead suggestive SNPs common in METAL and MRMEGA meta-analyses.

After cohort-specific quality controls (Supplementary Methods) and data harmonization, 27,217,987 variants met the inclusion criteria, and 2,970,850 were present in all cohorts. These common variants were used for further analyses. Both METAL- and MR-MEGA-based observed scale LD score regression heritabilities indicated low percentage of genetically explained variance (h_METAL_^2^=0.1184; h_MR–MEGA_^2^=0.085), with relatively high standard errors (SE_METAL_=0.051; SE_MR–MEGA_=0.057; Supplementary Table A3). Neither METAL nor MR-MEGA identified genome wide significant (p < 5×10^−8^) variants (Fig. 1(a)–(b), Fig. 2(a)–(b)). With less stringent level, p < 1×10^−5^, and combining results for METAL and MR-MEGA meta-analyses, we identified 23 suggestive genomic loci (Table 2). METAL identified 15 (Supplementary Table B1), while MR-MEGA 16 genomic loci (Supplementary Table B2), with eight genomic loci identified by both methods (Table 3). These eight suggestive genomic loci were located on RBM6:RBM5 (rs2013208), SOX5 (rs77480549), FAM110B (rs75065017), ZIC1 (rs9990273), PIK3CG (rs185087), SLIT3 (rs1875972), PLD1 (rs13083025) and C12orf55 (rs7137724) genes (Table 3, Supplementary Fig.B1(a)-(p)). Six of them showed homogeneous effects across participating cohorts (I^2^∈[0,58.2]; Table 3, Fig. 3, Supplementary Fig.B2). The other two - rs1875972 and rs7137724 - showed ancestrally heterogeneous effects, with significant ancestral heterogeneity (P_anc.het_≤0.0459), but not significant residual heterogeneity (P_res.het_≥0.2747, Table 3), suggesting population specific differences rather than other confounding factors.

**Figure 4.**
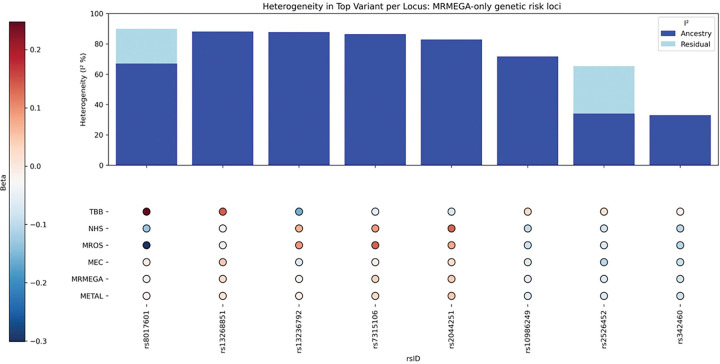
Heterogeneity in lead suggestive SNPs identified exclusively in MRMEGA meta-analyses.

**Figure 5.**
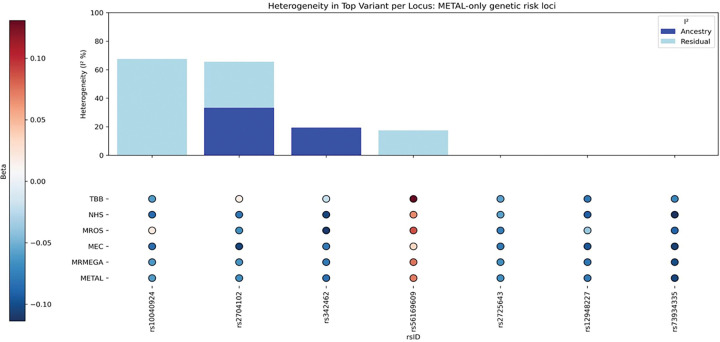
Heterogeneity in lead suggestive SNPs identified exclusively in METAL meta-analyses.

Among eight other genomic loci identified exclusively by MR-MEGA, six (Table 2) showed significant ancestry-related heterogeneity but not residual heterogeneity (Table 2; Fig. 4) with opposite effect directions in the TBB cohort versus all other cohorts (Supplementary Fig.A3(a)-(d)) or effect directions in TBB and MEC opposite to NHS and MrOS (Supplementary Fig.B3(e)-(g)). As opposed to this, genomic loci identified exclusively with METAL exhibited more homogeneous effects across study cohorts (Supplementary Table B3, Fig. 5, Fig.B4).

### Functional characterization of suggestive genomic loci

Two of our 23 identified genomic loci - rs2013208 and rs185087 - were previously reported in the literature. The one with the lowest p-values in both meta-analyses, rs2013208 associated with lower aMT6s, was previously associated with high HDL levels and coronary artery disease (CAD)^26^, as well as with sex differences in serum lipid profiles^27^. The other one, rs185087 (PIK3CG) also associated with lower aMT6s, was previously identified as increasing CAD risk^28^. When looking at the nearest protein coding gene to each suggestive lead SNP, genes with low probability of being loss-of-function intolerant (pLI) were found both in loci with (C12orf55, NEK6) and without (PLD1, COL25A1, MYH13, FAM110) significant ancestral heterogeneity (Table 2). We observed four genes with the pLI > 0.99 (RBM6: RBM5, ACTN1, SOX5 and SLIT3) and one (ZIC1) with pLI = 0.825 (Table 2). Genomic loci placed on these five genes with high pLI were identified as suggestive by both METAL and MR-MEGA.

### MAGMA gene set and tissue expression analysis

We used Functional Mapping and Annotation (FUMA^22,23^) to functionally annotate results of our meta-analyses. We ran multi-marker analysis of genomic annotation (MAGMA^29^) for gene ontology, tissue level and single cell expression data. For both METAL and MR-MEGA results, input SNPs were mapped to 15,857 protein coding genes and the genome-wide significance was defined at P = 3.153×10^−6^. No gene ontology terms were significant at this level for METAL (Supplementary Table B5), whereas for the MR-MEGA, “gomf_adenyl_nucleotide exchange_factor_activity” gene set was significant (adjusted P_bon_=0.021; Supplementary Table B6). Tissue expression analyses did not indicate any significant gene expression, neither with METAL nor with MR-MEGA (Supplementary Figs.B5-B6). Pathway enrichment tests implemented in GENE2FUNC did not indicate any significantly enriched differentially expressed genes (P_bon_<0.05) in METAL or MR-MEGA (Supplementary Fig. B7). At the gene set level, however, both methods indicated significant enrichment of input genes in positional gene set chr3p21 (P_MR–MEGA_=1.81×10^−19^; P_METAL_= 1.59×10^−18^, Supplementary Tables B4-B5, Supplementary Fig.B11), canonical pathways (MsigDB c2) in M22069 (“biocarta_msp_pathway”; P_MR–MEGA_= 2.67×10^−2^; P_METAL_= 3.37×10^−2^, Supplementary Tables B4-B5) and M27744 (“reactome_signaling_ by_mst1”; adjusted P_MR–MEGA_= 2.67×10^−2^; P_METAL_= 3.37×10^−2^, Tables B4-B5) human gene sets^30^. Both METAL and MR-MEGA prioritized genes were enriched in 19 GWAS catalog reported gene sets with a substantial overlap between the two methods, representing gene sets associated with cognition, physical activity, sleep, mental health, brain morphology, socioeconomic factors, and medical conditions (Supplementary Tables B4-B5, Supplementary Fig.B12).

### Comparison with previously published results

A previous genome-wide association study (GWAS) analysis conducted on 2,373 individuals from the TBB cohort identified five suggestive loci (P < 5×10^−6^) associated with urinary Log(aMT6s:Cr)^15^. However, none of these loci were detected in our meta-analysis (Supplementary Table C1). Among these variants, rs142037747 (GALNT15), is exceptionally rare, particularly in non-East Asian populations (EAF = 0.0002 in EUR, EAF = 0.001 in remaining ancestries; Supplementary Table C4). Due to its low minor allele frequency (MAF < 1%), this variant was excluded during quality control from the NHS, MrOS, and MEC cohort data. Another variant, rs7571016 (GALNT13) was absent in the MEC cohort GWAS results, likely due to platform-specific genotyping (TaqMan Assay Panel). The remaining three suggestive variants, rs17681554, rs9645614 and rs6451653, exhibited high heterogeneity in effect estimates across the study cohorts (Supplementary Table C5) with the TBB cohort effects differing markedly from those observed in other cohorts (Supplementary Fig.C1(a)-(e)). This suggest that previously reported aMT6s suggestive loci may be specific to East Asian ancestry (EAS), underscoring the need for larger ancestry-specific GWAS studies to validate these findings.

A recent meta-analysis^31^ (with a total sample of 8,011 individuals) of three European cohorts’ GWASs of 54 urinary metabolites identified three variants associated with Tryptophan (Supplementary Table C6), playing a key precursor role in melatonin production through the serotonin-melatonin pathway. These three variants were present in three of our cohorts each and were not significant in either METAL (Supplementary Table C7) or MR-MEGA (Supplementary Table C8) meta-analysis (p > 0.46). Also, no association was found in our study for any other of the 54 metabolites analyzed in Valo et.al^31^ (p > 0.06; Supplementary Table C9-C10).

### Polygenic risk score and PheWAS analyses

We used PRS-CSx (Supplementary Methods) on our meta-analysis cohorts’ data to estimate SNPs’ weights for the aMT6s polygenic risk score (PRS), and these weights were then applied to the UK Biobank and MGBB data to derive the aMT6s-PRSs. Ancestry-specific (EUR, EAS) and cross ancestry (META) PRSs were generated using ancestry-matched LD reference panels and SNPs with p < 0.05. Scores were calculated using PLINK^32^ and normalized (z-scored) within each ancestry group or across all ancestries for META-PRS.

Phenome-wide association studies (PheWAS) of aMT6s-PRSs were conducted in MGBB (EUR ancestry only) and UKBB (EUR, EAS, META; Supplementary Methods). In MGBB, of 1,657 disease outcomes tested, 101 associations were significant after Bonferroni correction (Supplementary Table D1). These included among others genitourinary (e.g. symptoms involving urinary system (p_adj = 3.6×10^−4^)), dermatologic (e.g. cellulitis (p_adj = 6.0×10^−3^)) endocrine/metabolic (e.g. obesity (p_adj = 2.5×10^−3^), type 2 diabetes (p_adj = 0.025)), circulatory system (e.g. chronic pulmonary heart disease (p_adj = 0.013)) or respiratory (e.g. septal deviations/turbinate hypertrophy (p_adj = 0.044)) diseases. Further, in the cross-ancestry UKB sample, both the EUR-PRS (Supplementary Table D2) and META-PRS (Supplementary Table D3) showed significant associations (Bonferroni corrected p < 0.05/1,509 = 3.3×10^−5^) with phenotypes including hereditary hemolytic anemias (p_adj = 1.3×10^−29^), sickle cell anemia (p_adj = 9.3×10^−22^), vitamin D levels (p_adj = 2.7×10^−44^), type 2 diabetes (p_adj = 9.8×10^−8^) and sleep duration (p_adj = 8.0×10^−9^). When restricted to European-ancestry UKB sample, only the META-PRS (Supplementary Table D4) was significantly associated with psoriasis and related disorders (p_adj < 0.017), while the EUR-PRS (Supplementary Table D5) and EAS-PRS (Supplementary Table D6) showed no significant associations.

Except from the disease outcomes included in the pheWAS analysis, we further examined associations of ancestry-specific (East Asian (EAS) and European (EUR)) and cross-ancestry (META) polygenic risk scores (PRSs) with four self-reported sleep related traits (sleep duration (in hours), short sleep duration, morningness and blood vitamin D levels; see Supplementary Tables D7-D13 for details). When all UK Biobank participants were included in the sample, both the z-scored East Asian-specific PRS (EAS-zPRS) and the European-specific PRS (EUR-zPRS) showed significant (Bonferroni adjusted for 3 PRSs×4 sleep traits; p < 0.05/12 = 0.004) associations with sleep duration (p < 2.81 × 10^−3^), short sleep duration (p < 7.97 × 10^−6^), blood vitamin D levels (p < 2.40 × 10^−6^), and morningness (p < 7.32 × 10^−7^) (Supplementary Table D7). The cross-ancestry PRS (META-zPRS) was significantly associated with all these traits except morningness (p = 0.716) (Supplementary Table D7). When we restricted to UK Biobank participants of European ancestry, the associations for the EUR-zPRS remained significant. In contrast, for the EAS-zPRS, only the association with morningness remained significant. For the META-zPRS, all associations except that with overall sleep duration remained significant (Supplementary Table D8). None of these associations remained significant when East Asian (EAS, Supplementary Table D9), Admixed American (AMR, Supplementary Table D11), Central/South Asian (CSA, Supplementary Table D12), or African (AFR, Supplementary Table D13) ancestry groups were used as the validation set. However, in the Middle Eastern (MID) group (Supplementary Table D10), the EAS-zPRS showed a significant association with blood vitamin D levels (p = 0.0012).

### Genetic correlation with sleep related traits in the UK biobank

We also used linkage disequilibrium score regression (LDSC) analysis to assess genetic correlations between our outcome measure and both self-reported and actigraphy-derived sleep traits in the UK Biobank, including relative amplitude^33^. This analysis revealed no strong genetic correlations (rg ranging from −0.3 to 0.2), and none reached statistical significance (smallest p = 0.09). (Table E1, Figure E1).

## Discussion

In this study, we conducted the largest to date GWAS meta-analysis of urinary 6-sulfatoxymelatonin (aMT6s) levels across five cohorts, including participants from diverse populations. Despite aggregating data from over 11,000 individuals, no genome-wide significant variants (P < 5 × 10^−8^) were detected, indicating that melatonin secretion is likely influenced by a complex polygenic architecture with small effect sizes. We identified 23 suggestive genomic loci, with 8 loci detected consistently across both METAL and MR-MEGA approaches. Two suggestive loci were previously implicated in coronary artery disease (rs2013208, rs185087) and one in lipid metabolism (rs2013208), suggesting potential pleiotropic effects linking melatonin metabolism to cardiometabolic pathways.

A recent large-scale GWAS of pineal gland volume^34^ identified 34 genome-wide significant loci and highlighted robust genetic contributions to melatonin-related neuroanatomy. Across both our melatonin metabolite GWAS and the pineal gland volume GWAS, several genes of interest overlap, pointing to shared biological mechanisms. The RBM6:RBM5 locus, which encodes RNA-binding proteins central to splicing regulation, emerges in both studies and highlights RNA processing pathways relevant to melatonin metabolism and pineal morphology. COL25A1, implicated in amyloid plaque formation and neurodegeneration, links melatonin biology to brain aging and disease vulnerability. Finally, signals in the ZIC gene family (ZIC1 in our study, ZIC4 in the pineal gland study) underscore the contribution of neurodevelopmental transcription factors to melatonin-related traits. Taken together, these convergences suggest common genetic pathways influencing both structural and metabolic aspects of melatonin biology.

A central finding of our study is the presence of significant ancestry-related heterogeneity in genetic associations. Several loci exhibited opposing effects across populations, particularly between the Taiwan Biobank (EAS) and the other cohorts. This highlights the potential population-specific genetic influences on melatonin metabolism and underscores the necessity for ancestry-stratified GWAS analyses. Our study did not replicate the five suggestive loci previously identified in the Taiwan Biobank GWAS. The rarity of some of these variants (e.g., rs142037747 in GALNT15) in non-East Asian populations contributed to their absence in our meta-analysis. Additionally, the heterogeneity in effect estimates across cohorts suggests that genetic influences on aMT6s levels may not be entirely shared across populations.

Gene set enrichment analysis identified significant pathways related to nucleotide exchange factors^35^, cellular signaling^36^, mitochondrial and energy regulation^37^ and cancer^38^. We also identified the MST1 signaling being key regulator of cell death, immune function, metabolism, and tumor suppression^39,40^. MST1 is degraded in breast cancer cells, reducing its tumor-suppressive activity^41^ and regulates YAP/TAZ inhibition leading to tumor suppression in prostate cancer^42^. MST1 activation is also detrimental in cardiovascular disease, promoting cardiomyocyte apoptosis, oxidative stress, and mitochondrial dysfunction^43^, leading to heart failure^44^ and myocardial infarction progression^45^. Inhibition of MST1 emerges as a potential therapeutic strategy to protect against heart disease. Furthermore, the identified significant MSP (Macrophage Stimulating Protein) pathway, plays a crucial role in cell migration and wound healing^46^, immune regulation^47^, cancer progression^48,49^, cancer invasion and metastasis^50^ and excessive inflammation suppression^51,52^. The integration of these pathways suggests that melatonin may exert protective effects by modulating critical processes such as nucleotide exchange, cell survival, and immune regulation. Targeting MST1 and MSP pathways could provide novel therapeutic avenues for both cardiovascular diseases and cancer, supporting the multifaceted role of melatonin in human health.

Our polygenic risk score (PRS) analysis demonstrated associations between melatonin PRS and both blood vitamin D levels and sleep duration. These findings are consistent with prior evidence suggesting an interaction between melatonin and vitamin D metabolism and further underline the genetic link between melatonin secretion and sleep regulation.

Our study has several limitations. First, the overall heritability estimates suggest that aMT6s levels have a relatively modest genetic component, which may require larger sample sizes to detect genome-wide significant associations. Second, differences in sample collection, urine processing, and melatonin measurement methodologies across cohorts may have introduced residual variability, despite our efforts to normalize and harmonize the data. In addition, raw melatonin levels varied across cohorts, reflecting cohort-specific characteristics (e.g., particularly low levels among older MrOS men and much higher levels among NHS women). Although we adjusted for creatinine levels, applied logarithmic transformation, and standardized the data (z-scoring), these steps only partially mitigated the between-cohort differences. Together with the inclusion of multiple ancestries and relatively modest sample sizes, these factors likely contributed to the lack of genome-wide significant associations. Future studies should focus on expanding sample sizes, particularly in non-European populations, to improve power and capture ancestry-specific effects.

In conclusion, this study represents one of the largest multi-ancestry GWAS meta-analyses of urinary aMT6s levels to date, providing novel insights into the genetic determinants of melatonin secretion. While no genome-wide significant loci were identified, we uncovered several suggestive genetic signals with potential links to cardiometabolic traits and cancer. The observed ancestry-specific effects highlight the complexity of melatonin metabolism and the importance of diverse study populations. Future research should aim to replicate and extend these findings using larger and more diverse cohorts, ultimately improving our understanding of melatonin’s role in human health.

## Methods

### Study sample

The analytic sample for our study was made up of 11,744 individuals (Table 1) with 2,373 individuals of East Asian ancestry from the Taiwan Biobank (TBB), 3,861 female nurses of European ancestry from the Nurses’ Health Study (NHS1) and Nurses’ Health Study II (NHS2), 2,175 men of European ancestry from the Osteoporotic Fractures in Men (MrOS), and 3,335 individuals of diverse ancestries from the Multiethnic Cohort Study (MEC). Further details on each study cohort can be found in Supplementary Methods.

### Outcome definition

Table 4 summarizes the aMT6s measurement details across participating cohorts, with additional cohort-specific information provided in the Supplementary Methods. The concentration of melatonin metabolite (6-sulfatoxymelatonin, aMT6s) in the urine —whether measured from a first-morning void or an overnight collection— depends on the sample’s volume, with urinary creatinine levels serving as a reliable substitute for this variation^16^. The melatonin-to-creatinine ratio (aMT6s:Cr) is conventionally used to control for differences in sample dilution, with further log-transformation ensuring normality. To address inter-study variability, we additionally normalized (z-scored) the log-transformed aMT6s:Cr values within each study cohort, thereby establishing our primary outcome measure as Zlog(aMT6s:Cr).

### Genome-Wide Association and Meta-Analysis

Genome wide association studies (GWASs) of our outcome variable were performed within each study cohort using linear regression models adjusted for age at sample collection, sex (where applicable) and first 10 principal components of ancestry. Further cohort specific details on GWAS analyses are described in Supplementary Methods. GWAS summary statistics were aligned to genome build hg19 and harmonized by excluding variants with poor imputation quality (info score R^2^< 0.7), low minor allele frequency (MAF<1%), significant deviation from the Hardy-Weinberg equilibrium (p-value< 10^−8^) and the low genotype call rate (<95%). Meta analyses were performed by using meta-regression of multi-ethnic genetic association (MR-MEGA^17^) and inverse variance fixed-effects model in METAL^18^ with genomic control correction for the individual study level data. Briefly, MR-MEGA conducts a meta-regression analysis by creating axes of genetic variation specific to each cohort, which are next used as covariates in the meta-analysis to adjust for possible differences in population structure. This approach allows to distinguish between ancestral and residual sources of heterogeneity. Although MR-MEGA shows an increased power to detect SNPs associations under both fixed and random effects meta-analysis settings^17^, for variants with homogenous effects across populations its power is reduced^19^. To account for these aspects and potentially detect genetic variants with both homogeneous and heterogeneous effects across study cohorts, we applied both METAL and MR-MEGA methods. To claim significance, we applied the conventional genome-wide threshold of 5´10^−8^ and less stringent level of 1´10^−5^ for suggestive associations^20^. The overall SNP-based heritability and genetic correlations were estimated by using Linkage Disequilibrium Score Regression (LDSC)^21^. To identify independent significant genomic risk loci we used Functional Mapping and Annotation (FUMA^22,23^) v1.5.2. We further derived a polygenic risk score for the aMT6s using PRS-CSx^24^ and checked its associations with common diseases by running phenome-wide association studies (PheWASs) in the Mass General Brigham Biobank (MGBB) and in the UK Biobank (UKBB). Further details are described in Supplementary Methods.

## Supplementary Material

Supplementary Files

This is a list of supplementary files associated with this preprint. Click to download.

• SupplementaryMethodsclean.docx

## Figures and Tables

**Table 1:** Descriptive characteristics of study participants with urinary 6-sulfatoxy melatonin and genetic data available in Taiwan Biobank (TBB), the Nurses' Health Study I (NHS1), the Nurses' Health Study II (NHS2), MrOs and Multiethnic Cohort (MEC).

	TBB	NHS*	MrOs	MEC	Total
**Sample size (n)**	2,373	3,861	2,175	3,335	11,744
Men; N (%)	890 (37.51)	0 (0)	2,175 (100)	3,335 (100)	6,400 (54.5)
Women; N (%)	1,483 (62.5)	3,861 (100)	0 (0)	0 (0)	5,344 (45.5)
Age in years, mean (SD)	50.8 (10.8)	57.6 (12.8)	73.1 (5.6)	68.9 (7.7)	62.3 (10.0)
BMI, mean (SD)	24.7 (5.8)	26.61(5.6)	27.4 (3.7)	26.5 (4.0)	26.3 (4.9)
**Ancestry/race**					
Black; N (%)	0 (0)	0 (0)	0 (0)	436 (13.1)	436 (3.7)
East Asian; N (%)	2,373 (100)	0 (0)	0 (0)	0 (0)	2,373 (20.2)
Japanese American; N (%)	0 (0)	0 (0)	0 (0)	1,494 (44.8)	1,494 (12.7)
Latino; N (%)	0 (0)	0 (0)	0 (0)	226 (6.8)	226 (1.9)
Native Hawaiian; N (%)	0 (0)	0 (0)	0 (0)	290 (8.7)	290 (2.5)
White; N (%)	0 (0)	3,861 (100)	2,175 (100)	889 (26.7)	6,925 (59)
**Outcome measures characteristics**					
**aMT6s (ng/mL)**					
Median (IQR)	20.41 (11.88; 30.19)	24.99	7.58	16.9	
Mean (SD)		44.3(5.65)	9.83 (7.98)	23.5 (23.9)	
**log(aMT6s/Cr)**					
Median (IQR)	2.83 (2.33; 3.31)		2.14	−1.45	
Mean (SD)		3.11 (1.16)	2.06 (0.85)	−1.59 (1.05)	

* NHS : pooled NHS1 and NHS2 cohorts.

**Table 2:** Meta analyses results for lead SNPs. Bolded are i) lead variants with p values < 10–5 (suggestive) in meta-analyses by both METAL and MRMEGA ii) p values < 10–5 (suggestive) from association tests; iii) p-values for heterogeneity tests < 0.05 (significant); iv) pLI scores > 0.99 (putative causal) and v) effect allele frequencies (EAF) in the cohort with the highest EAF.

Lead SNP							MRMEGA					METAL				pLI	EAF			
GL	rsID	Nearest Gene	CHR	BP	NEA	EA	Effect	StdErr	P.value	Panc.het	Pres.het	Effect	StdErr	P.value	P.Het		TBB	NHS	MrOS	MEC
1	**rs2013208**	RBM6:RBM5	3	50129399	C	T	−0.065	0.020	**4.26E-07**	9.54E-02	0.181	−0.074	0.014	**2.84E-07**	1.04E-01	**1.000**	**0.858**	0.496	0.514	0.679
2	**rs7137724**	C12orf55	12	97265256	C	T	0.057	0.010	**5.20E-07**	**2.44E-03**	0.676	0.070	0.016	**9.87E-06**	**1.93E-02**	0.000	**0.849**	0.717	0.722	0.811
3	rs342460	AFF1	4	88059242	A	G	−0.078	0.010	**5.76E-07**	5.24E-02	0.697	−0.083	0.017	**6.76E-07**	2.15E-01	0.477	0.181	0.201	**0.210**	0.173
4	**rs8017601**	ACTN1	14	69380814	A	G	−0.040	0.037	**1.22E-06**	**2.12E-07**	0.225	−0.016	0.030	5.91E-01	**1.56E-06**	**1.000**	0.051	0.037	0.034	**0.084**
5	rs13268851	PVT1	8	129100295	G	A	0.026	0.003	**1.79E-06**	**4.97E-07**	0.952	0.015	0.014	2.77E-01	**1.37E-05**	*	0.288	**0.406**	**0.406**	0.326
6	**rs2526452**	COL25A1	4	110103895	C	G	−0.065	0.024	**2.45E-06**	7.33E-02	0.065	−0.067	0.014	**2.37E-06**	**3.45E-02**	0.000	**0.738**	0.675	0.683	0.681
7	**rs77480549**	SOX5	12	24107330	T	C	−0.123	0.031	**2.53E-06**	8.90E-01	0.460	−0.119	0.024	**4.24E-07**	6.69E-01	**0.999**	0.**286**	0.013	0.014	0.146
4	rs372896	RP11-723P16.3	14	69324446	A	C	0.029	0.025	**4.52E-06**	**3.97E-06**	0.457	0.051	0.028	6.84E-02	**4.57E-05**	*	0.321	0.324	0.333	**0.373**
8	**rs1875972**	SLIT3	5	168288992	G	A	−0.060	0.016	**4.62E-06**	4.59E-02	0.275	−0.063	0.014	**6.11E-06**	9.05E-02	**0.992**	0.127	0.127	0.133	**0.139**
9	rs13236792	XRCC2	7	152394108	T	C	−0.016	0.007	**5.04E-06**	**8.49E-07**	0.871	−0.008	0.020	6.97E-01	**2.10E-05**	*	**0.396**	0.184	0.180	0.288
10	rs7315106	RP11-405A12.2	12	20025120	G	A	0.037	0.013	**6.19E-06**	**5.76E-06**	0.478	0.029	0.016	6.57E-02	**6.89E-05**	*	**0.366**	0.258	0.258	0.249
11	rs10986249	NEK6	9	126918060	C	G	−0.054	0.010	**7.20E-06**	**1.81E-03**	0.644	−0.055	0.015	2.07E-04	1.42E-02	0.039	0.066	0.257	**0.264**	0.177
12	**rs185087**	PIK3CG	7	106489559	T	C	−0.062	0.029	**8.12E-06**	9.56E-02	0.104	−0.077	0.017	**5.91E-06**	6.62E-02	0.000	**0.184**	0.140	0.146	0.160
13	rs2044251	AC091736.1	7	135507742	A	T	0.046	0.013	**8.18E-06**	**4.77E-05**	0.606	0.048	0.018	8.99E-03	**5.65E-04**	*	0.049	**0.080**	0.078	0.068
14	**rs75065017**	FAM110B	8	59081401	A	G	−0.130	0.019	**8.57E-06**	3.48E-01	0.608	−0.123	0.026	**2.46E-06**	6.01E-01	0.173	**0.500**	0.412	0.412	0.360
15	**rs9990273**	ZIC1	3	147267582	G	C	0.061	0.014	**8.95E-06**	2.46E-01	0.357	0.062	0.013	**3.28E-06**	3.37E-01	0.825	0.248	**0.437**	**0.437**	0.314
16	**rs13083025**	PLD1	3	171487632	G	C	0.059	0.014	**9.79E-06**	1.08E-01	0.363	0.063	0.014	**6.95E-06**	2.05E-01	0.000	**0.858**	0.496	0.514	0.679
17	rs342462	AFF1	4	88057481	G	A	−0.101	0.010	**5.85E-07**	8.23E-01	0.886	−0.083	0.017	**5.90E-07**	2.93E-01	0.477	0.174	0.200	0.209	0.178
18	rs56169609	AC007204.2	19	20091038	A	C	−0.079	0.006	1.06E-05	6.18E-02	0.884	0.072	0.015	**2.01E-06**	3.04E-01	*	0.136	0.358	**0.375**	0.224
19	rs2704102	COL25A1	4	110103485	C	T	−0.065	0.024	**2.50E-06**	8.26E-02	0.056	−0.067	0.014	**2.19E-06**	**3.31E-02**	0.000	**0.738**	0.683	0.683	0.682
20	rs2725643	RP11-281H11.1	8	5552149	A	G	−0.062	0.029	2.85E-05	4.99E-01	**0.012**	−0.067	0.015	**5.42E-06**	9.29E-01	*	0.240	**0.316**	0.312	0.304
21	rs10040924	HMGB1P29	5	123466039	C	T	−0.067	0.007	2.44E-05	1.00E + 00	0.795	−0.061	0.014	**5.70E-06**	**2.62E-02**	*	**0.628**	0.500	0.521	0.604
22	rs12948227	MYH13	17	10265366	C	T	−0.080	0.017	2.89E-05	7.23E-01	0.429	−0.079	0.017	**5.71E-06**	6.16E-01	0.000	0.117	0.224	**0.225**	0.144
23	rs73934335	AC127383.1	2	68657017	G	A	0.075	0.021	3.88E-05	6.64E-01	0.176	−0.104	0.023	**7.45E-06**	9.62E-01	*	0.026	**0.130**	0.120	0.077

GL-genomic loci, CHR-Chromosome, BP-base pair, NEA-non-effect allele, EA-effect allele, Effect-association test effect size estimate, StdErr-standard error of the effect size estimate, P.value-p value for the two sided association test, Panc.het-p value for the two sided ancestral heterogeneity test (chi-square test with 1df), Pres.het-p value for the two sided residual heterogeneity test (chi-square test with 2df), P.Het - p value from the two sided test for heterogeneity (chi-square test with 3df), pLI - probability of being loss-of-function-intolerant for the nearest coding gene, EAF - effect allele frequency, TBB-Taiwan Biobank, NHS - merged NHS1 and NHS2 cohorts, MEC - Multiethnic cohort;

* pLI score was not available for this gene (gnomAD v2.1.1)

**Table 3:** Common suggestive genomic loci identified in meta-analysis of Zlog(aMT6s:Cr) by both METAL and MR MEGA, with association and heterogeneity statistics

Suggestive genomic loci					MRMEGA					METAL				
	rsID	chr	pos	nearest Gene	P.value	beta	se	Panc.het	P res.het	P.value	beta	se	I2	P. Het.
1	rs2013208	3	50129399	RBM6:RBM5	**4.26E-07**	−0.0654	0.0199	0.0954	0.1805	**2.84E-07**	−0.0739	0.0144	51.4	0.1036
2	rs77480549	12	24107330	SOX5	**2.53E-06**	−0.1226	0.0308	0.8902	0.4600	**4.24E-07**	−0.1192	0.0236	0	0.6694
3	rs75065017	8	59081401	FAM110B	**8.57E-06**	−0.1296	0.0190	0.3481	0.6082	**2.46E-06**	−0.1230	0.0261	0	0.6010
4	rs9990273	3	147267582	ZIC1	**8.95E-06**	0.0613	0.0136	0.2456	0.3565	**3.28E-06**	0.0623	0.0134	11.3	0.3365
5	rs185087	7	106489559	PIK3CG	**8.12E-06**	−0.0623	0.0289	0.0956	0.1044	**5.91E-06**	−0.0773	0.0171	58.2	0.0662
6	rs1875972	5	168288992	SLIT3	**4.62E-06**	−0.0604	0.0159	**0.0459**	0.2747	**6.11E-06**	−0.0632	0.0140	53.7	0.0905
7	rs13083025	3	171487632	PLD1	**9.79E-06**	0.0585	0.0143	0.1080	0.3629	**6.95E-06**	0.0630	0.0140	34.6	0.2045
8	rs7137724	12	97265256	C12orf55	**5.20E-07**	0.0568	0.0102	**0.0024**	0.6764	**9.87E-06**	0.0698	0.0158	69.8	**0.0193**

P.value - p value for a SNP from the association test; beta - SNP’s effect size estimate; se - standard error of the SNP’s effect size estimate; I2 -I^2^ Statistics: percentage of variation in the SNP’s effect size across studies due to heterogeneity; P.Het. - p value for Cochrane heterogeneity test; P anc.het- p value for the ancestry heterogeneity test; P res.het - p value for the residual heterogeneity test.

## Data Availability

The datasets used and analyzed during the current study are available from the corresponding author on reasonable request.
